# The Case of President Garfield

**Published:** 1881-11

**Authors:** 


### THE CASE OF PRESIDENT GARFIELD.

WE regret to find that certain medical men are disposed to criticise the course pursued by the physicians who attended President Garfield.

The autopsy proved beyond all doubt that the wound, from the instant it was inflicted, was necessarily fatal, and would have been fatal in spite of anything that could have been done, even if a correct diagnosis of the case had been possible immediately after the injury. The autopsy further proved the wisdom of the surgeons when they decided not to cut for the ball, for it had become completely encysted and would have done no harm to the patient, whereas had an effort been made to extract the ball, the operation would have been a painful one, which, if completed, would have resulted in almost immediate death.

The judgment of all fair-minded men will be that the medical and surgical treatment of the eminent patient was wise and judicious, and that all the appliances for his comfort were as near perfect as possible.

It seems to us that it would be wiser for these medical critics to gracefully acknowledge that they were mistaken than to endeavor to keep up the fight when the facts are against them.

I was a student of Dr. Frank H. Hamilton, and interne under him in the Buffalo hospital. From my knowledge of the man I felt that a wise thing had been done when he was called to Washington; but I confess I felt disappointed when he advised that no extensive exploration should be made for the bullet; but I trusted to his judgment, and as usual I found that it was correct.

---

CAPACITY OF CATHEDRALS AND CHURCHES.—In Forbes' *Tourists* the capacity of the larger European churches and cathedrals is given as below : St. Peter's Church, Rome, holds 54,000 people ; St. Paul's, London, 35,000 ; St. Sophia's, Constantinople, 33,000 ; the Florence Cathedral, 24,300 ; St. Petronius, Bologna, 24,000 ; St. Paul's, Rome, 32,000 ; St. John Lateran, 22,900 ; Notre Dame, Paris, 20,000 ; the Pisa Cathedral, 13,000 ; St. Stephen's, Vienna, 12,400 ; St. Dominico, Bologna, 2,000 ; St. Peter's, Bologna, 11,500 ; the Cathedral of Vienna, 11,000 ; St. Mark's, Venice, 7,000 ; the Milan Cathedral, 7,000. These figures, it will be remembered, do not refer to seating capacity.

